# Sjögren’s Syndrome is Rare in Psoriasis, but Common in Psoriatic Arthritis: Two Sides of a Coin – A Single Centre Retrospective Study

**DOI:** 10.31138/mjr.270224.tcs

**Published:** 2025-05-28

**Authors:** Kerem Abacar, Şeyma Çolakoğlu-Özkaya, Tülin Ergun, Alida Aliyeva, Seda Kutluğ-Ağaçkiran, Gizem Sevik, Seher Küçükoğlu-Cesur, Mehmet Soy, Pamir Atagündüz

**Affiliations:** 1 Division of Rheumatology, Department of Internal Medicine, Marmara University School of Medicine, Istanbul, Turkey,; 2 Department of Medical Biology and Genetics, Institute of Health Sciences, Marmara University, Istanbul, Turkey,; 3 Department of Dermatology, Marmara University School of Medicine, Istanbul, Turkey,; 4 Department of Dermatology, School of Medicine, Altinbaş University, Istanbul, Turkey,; 5 Division of Rheumatology, Department of Internal Medicine, School of Medicine, Altinbas University, Istanbul, Turkey

**Keywords:** Sjögren’s syndrome, psoriasis, psoriatic arthritis, axial spondyloarthritis, anti-nuclear antibody, sicca symptoms

## Abstract

**Background:**

Sjögren’s syndrome (SS) may accompany psoriatic arthritis (PsA), if ever, and its presence during psoriasis (PsO) is rare. Recent observations of an association with predominantly axial forms of PsA are intriguing. Whether SS complicates PsO and PsA to the same extent is not known.

**Objectives:**

To investigate the frequency of SS in patients with PsO and PsA, and elucidate disease features associated with SS. Methods: In this retrospective analysis, we collected the data about previously recorded in the follow-up files of PsO and PsA patients. We classified patients who met the 2002 American-European Sjögren’s syndrome Consensus Group Criteria (AECG) or the 2016 ACR-EULAR Classification Criteria for primary Sjögren’s Syndrome as SS. Frequency rates of SS in PsA patients and in PsO patients without arthritis were compared and SS characteristics were described.

**Results:**

Of the 184 PsO patients, 112 had PsA and 72 PsO alone. A substantial proportion of PsA patients had SS. This association was not present in the PsO group (PsA: 20(%17.9), PsO: 0(%0), p<0.001). Gender distribution, mean age, and PsO onset age were similar in both. Symmetric polyarticular involvement was rare, and most PsO patients with arthritis were ANA positive at a serum dilution level of 1:320 and beyond.

**Conclusions:**

SS seems to be part of the clinical picture in a substantial proportion of PsA patients, and its absence in PsO implies that SS may be arthritis associated. Whether the type of arthritis is critical in developing SS in PsA deserves further studies with large populations.

## KEY MESSAGES

### What is already known about this subject?

Secondary Sjögren’s syndrome (sSS) may accompany autoantibody-driven diseases such as rheumatoid arthritis, systemic lupus erythematosus, and systemic sclerosis.

### What does this study add?

The higher frequency of Anti-Nuclear Antibody (ANA) positivity and secondary Sjögren’s syndrome in psoriatic arthritis (PsA) compared to psoriasis (PsO) suggest that B lymphocyte-driven immunopathogenic pathways may contribute to the emergence of PsA.

### How might this impact clinical practice?

Sjögren’s syndrome (SS) seems to be part of the clinical picture in a substantial proportion of PsA patients, and its absence in PsO implies that SS may be arthritis-associated.

## INTRODUCTION

Sjögren’s syndrome (SS) is a chronic systemic autoimmune disease that primarily affects the exocrine glands and induces xerostomia and keratoconjunctivitis sicca. Although the disease can be seen as primary SS (pSS), it may accompany many autoimmune diseases.^1^ The prevalence of sSS varies between 4% and 31% in rheumatoid arthritis (RA), 14% and 20.5% in systemic sclerosis (SSc) and 6.5% and 19% in systemic lupus erythematosus (SLE).^2^ The presence of autoantibodies such as Rheumatoid Factor (RF) and Anti-Nuclear Antibody (ANA) typical for RA and SLE in pSS implies similarities of pathogenesis, as well. Effective use of B cell-depleting therapies, such as rituximab, in these autoimmune diseases provides more evidence that B cells play a paramount role in the pathogenetic process.^3–6^

Previously, Kroese et al. reported the critical role of B cell hyperactivity in the pathogenesis of pSS in this context.^7^ B lymphocytes propagate the occurrence of the pathological hallmark of SS, the focal lymphocytic sialadenitis formation.^8^ The critical role of T-helper (Th) 17 cells as a triggering factor in the organisation of the relatively incoordinate lymphocytic infiltrate formed by a relative increase in B-lymphocytes in the salivary gland has been suggested in several reports.^9^ Additionally, the contribution of T-cells to local inflammation is supported further by the increased production of interleukin (IL) -17, IL-21, and interferon (IFN) gamma in the salivary gland substrates.^10^

The role of the B-cells in PsA pathogenesis is not fully understood. In this regard, the presence of autoantibodies is of special interest.^11^ Previous studies suggested that up to 40% of PsA patients tested positive for ANA, and the role of ANA in the pathogenesis of PsA is yet to be elucidated.^12^ Rosenberg et al. observed a higher rate of ANA positivity in PsA patients than in ankylosing spondylitis (AS) and especially in non-arthritic PsO.^13^

PsA is unique among inflammatory joint diseases with its multiple patterns of joint involvement. PsA may involve peripheral joints, axial joints, or both, and up to one-third of PsO patients will eventually develop PsA.^14^ PsA and psoriasis share immunopathologic mechanisms mediated through the Interleukin (IL) 23/IL17 pathway and Tumour Necrosis Factor (TNF) alpha.^15^ However, distinct organ-specific targets involved in both entities imply deviations from *shared* pathogenesis. Similarly, the diverse effects^16^ of anti-cytokine therapies in these clinically differing patient groups suggest that organ involvement may occur through different immunological pathways in non-arthritic PsO and PsA. In this regard, recent awareness of the sicca symptoms and SS in association with spondyloarthritis (SpA), especially with PsA but not with PsO, is interesting.^17^

Similarly, a previous study with small sample size reported a decrease in salivary flow rate in 60% of PsA and 16% in PsO; and the dry eye was present in 23% of PsA and 16% in PsO.^18^ The coexistence of PsO and SS is supported with some case reports, only.^19–21^ In the absence of solid data, physicians tend to treat skin and sicca symptoms similarly when PsO and SS coexist, due to the supposedly shared cytokine pathways in the pathogenesis.^22^

In this study, we aimed to investigate the prevalence of SS in both PsO and PsA and compare clinical and laboratory features of patients with and without SS in a large patient group.

## MATERIAL AND METHODS

### Patients

The clinical follow-up files of PsA patients who were already followed up in the rheumatology clinic and fulfilled CASPAR criteria,^23^ and the clinical follow-up files of PsO patients who were already followed up in the dermatology clinic were screened retrospectively. All the patients are examined at three-month intervals during their follow-up. Thereafter patients whose information regarding dry mouth and eyes (positive or negative) was already recorded in the clinical follow-up file were included in the analysis. Patients for whom sicca symptom information was not available according to the 2002 American-European Sjögren’s syndrome Consensus Group Criteria (AECG) excluded from analysis.^24^

### Sicca findings

To objectively detect subjective sicca symptoms in both rheumatology and dermatology clinics, Schirmer’s test is routinely performed on patients with dry eyes, and salivary gland scintigraphy and unstimulated salivary flow rate (U-SFR) are also routinely performed on patients with dry mouth. Schirmer’s test, salivary gland scintigraphy, and the unstimulated salivary flow rate (U-SFR) data of the patients with sicca symptoms were recorded retrospectively. Saliva is collected for 15 minutes, and the total amount was recorded in millilitres. A cut-off value for low salivary flow rate of ≤0.1 ml/min is used.^25^ However, patients using drugs affecting saliva (acitretin, pilocarpine, cevimeline) were excluded.

### Sjögren’s syndrome classification

In our clinic, every patient with objective dry mouth and eyes is investigated for SS. In this context, minor salivary gland (MSG) biopsy and laboratory tests are routinely performed. The classical histopathologic lesion of MSG is an infiltrate of ≥50 lymphocytes called foci. The number of foci per 4 mm2 is calculated into a focus score (FS). An FS of ≥1 is regarded as a positive finding for MSG histopathology.^24^ The results of previous ANA, RF, Anti-SSA, and Anti-SSB antibody tests of all PsO and PsA patients were recorded. Additionally, complement and immunoglobulin levels of SS-classified patients, if available, were recorded retrospectively. Patients who met the 2016 ACR-EULAR Classification Criteria for primary Sjögren’s Syndrome or the 2002 AECG criteria with all findings and diagnostic tests were classified as SS.^24,26^ Chronic viral infections, sarcoidosis and infiltrative diseases of the lacrimal gland were ruled out in all study patients.

### Systemic organ involvement and patterns of arthritic involvement

Data of constitutional, respiratory, cardiac, central or peripheral neuropathic symptoms, lymphadenopathy, and parotitis attacks of all SS patients throughout the entire follow-up period were collected from clinical follow-up files retrospectively.

For classifying as axial involvement, pelvis radiographs of the patients that have already performed were evaluated according to the 1984 Modified New York criteria, and sacroiliac Magnetic Resonance Imaging (MRI) images that have already performed were also assessed for active sacroiliitis according to ASAS definitions retrospectively.^27,28^ Data for HLA-B27 and subtypes of PsA were obtained from patient files. For axial disease classification, ASAS classification criteria for axial SpA were used.^29^

### Statistical Analyses

Data were analysed using Statistical Package for the Social Sciences 22.0 (SPSS, Chicago, IL, USA). The continuous variables were expressed as mean (standard deviation) and median (interquartile range (IQR)) for standard and non-normal distribution, respectively. Frequency (%) was used for the description of categorical variables. The comparison of variables was evaluated by the Mann Whitney U test, independent-samples t-test, Wilcoxon test, or chi-square test.

Approval for this study was obtained from the Marmara University Faculty of Medicine local ethics committee (09.2023.416).

## RESULTS

One hundred and twelve PsA and 72 PsO patients’ data were included in the final analysis. The main age in PsA and PsO patients was similar (48.3±11.9 and 49.4±13.5, respectively; p=0.59). Similarly, female to male ratio (rates (%) for PsO: 43/29 (59.7/40.3) and PsA: 82/30 (73/27), respectively; p= 0.08) and age of onset of psoriatic skin changes (mean (SD) for PsO: 30.3 ±10.9, and PsA: 29.4 ±13.2, respectively; p=0.68) did not differ between PsO and PsA patients. The follow-up period of PsA patients in the rheumatology clinic was mean (SD): 11.2 (3.4) years. None of the patients were under other specific treatments that could potentially cause sicca symptoms.

SS was detected in PsA patients only (PsA: 20/112 (%17.9), PsO: none, p<0.001). Out of 26 patients with oral and ocular dryness symptoms (24 patients were PsA and two were PsO), 20 patients met the 2002 AECG criteria for secondary SS. 70 PsO patients and 88 PsA patients had no sicca symptoms. Eighteen patients had salivary gland involvement detected in salivary gland scintigraphy, six Anti-SSA positivity, and five low U-SFR. All PsA patients with SS had a positive Schirmer’s test. MSG biopsy was performed in 13 patients with sicca symptoms. Biopsy findings of seven patients were found to be compatible with SS (**Table 1**).

**Table 1.** Characteristics of patients with PsA/SS coexistence.

	**Age**	**Gender**	**PsO-onset Age**	**Arthritis-onset Age**	**Axial Disease**	**HLA-B27**	**Radiographic sacroiliitis**	**Achilles enthesitis**	**Plantar fasciitis**	**Salivary Gland Scintigraphy**	**Schirmer's test positivity**	**U-SFR positivity**	**ANA positivity**	**ANA-titer**	**Anti-Ro positivity**	**Biological therapy**	**FS≥1 on MSG biopsy**	**pSS (ACR/EULAR 2016 criteria)**
Patient 1	69	F	54	67	yes		no	yes	yes	yes	yes	no	yes	1/100	no	no	no data	no
Patient 2	36	F	28	33	no		no	no data	no data	yes	yes	no	yes	1/320	yes	no	no data	yes
Patient 3	66	F	65	42	no		no	yes	no	yes	yes	no	yes	1/320	no	TNFi	yes	Yes
Patient 4	79	F	43	39	no		no	no	no	yes	yes	no data	no		no	no	no data	No
Patient 5	49	F	26	45	no		no	no	no	yes	yes	no	no data		no	secukinumab	yes	yes
Patient 6	68	F	48	48	no		no	yes	yes	yes	yes	no data	no		no	no	no	No
Patient 7	73	F	27	46	yes	negative	yes	no	no	yes	yes	yes	no		no	ustekinumab	yes	Yes
Patient 8	66	F	36	40	no	negative	no	no data	no data	yes	yes	yes	yes	1/640	yes	no	no data	Yes
Patient 9	55	F	38	45	yes	negative	no	no	no	no data	yes	yes	no		no	TNFi	no	No
Patient 10	61	F	29	59	no		no	no	no	yes	yes	yes	yes	1/320	no	risenkizumab	yes	Yes
Patient 11	36	F	21	36	yes	positive	no	no	no	yes	yes	no	yes	1/100	yes	secukinumab	no data	Yes
Patient 12	47	F	38	36	yes	positive	yes	no data	no data	yes	yes	no	no		no	no	no	No
Patient 13	50	F	43	48	no		no	no	no	no data	yes	no	yes	1/320	yes	secukinumab	no data	yes
Patient 14	32	F	28	31	no		no	no data	no data	yes	yes	yes	yes	1/320	yes	no	no data	yes
Patient 15	47	M	13	43	no		no	yes	no	yes	yes	no	no		no	no	yes	No
Patient 16	61	F	9	45	no		no	no	yes	yes	yes	no	no		no	no	no	No
Patient 17	48	M	16	45	no		no	no	yes	yes	yes	no	no		no	no	no	No
Patient 18	45	F	16	32	yes	negative	no	yes	yes	yes	yes	no data	yes	1/100	no	TNFi	yes	Yes
Patient 19	34	F	19	32	no		no	no	no	yes	yes	no	yes	1/100	yes	secukinumab	no	Yes
Patient 20	56	F	15	39	no		no	no	no	yes	yes	no	no		no	TNFi	yes	Yes

PsO: psoriasis; U-SFR: unstimulated salivary flow rate; ANA: Anti-Nuclear Antibody; SS: Sjögren’s syndrome.

Systemic organ involvement or lymphoma associated with SS was not observed in any of the PsA with the SS patients. While hypocomplementemia was detected in only three patients (15%), hypergammaglobulinemia or parotitis attack was not observed in any of the SS patients.

Type of articular involvement favored axial SpA and asymmetric oligoarticular arthritis and symmetric polyarticular PsA was present in about one third of these patients. PsA with SS patients were significantly more positive for ANA and had plantar fasciitis more frequently than PsA patients without SS (**Table 2**). Plantar fasciitis and Achilles enthesitis of the patients were defined by clinical examination and x-ray images only. However, the number of evaluated patients in the patient files was limited. Although not statistically significant, the age of onset of joint involvement in SS/PsA patients was higher and was treated less with biological therapy (**Table 2**).

**Table 2.** Comparison of clinical and laboratory features of patients with and without Sjögren’s syndrome.

	**Presence of SS**	**Absence of SS**	**p**
	**n=20**	**n=92**	
Age, mean, years (SD)	53.9 (11.1)	48.4 (10.7)	0.09
Female/Male (n)	18/2	64/28	0.08
Age of onset of skin involvements, years, mean (SD)	30.6 (6.4)	29.4 (6.2)	0.74
Age of onset of joint involvements, years, mean (SD)	42.6 (8.7)	37.4 (7.7)	0.09
Axial Involvement, n (%)	6 (35.3)	25 (29.4)	0.77
Sacroiliitis on MRI, n (%)	5 (29.4)	21 (24.7)	0.69
Sacroiliitis on X-Ray, n (%)	3 (17.7)	13 (15.3)	0.81
Polyarticular involvement, n (%)	4 (22.2)	11 (14.9)	0.29
Heel Enthesitis, n (%)	5/16* (31.3)	12/56* (21.4)	0.19
Plantar Fasciitis, n (%)	5/16* (31.3)	6/67* (9)	***0.03***
Erosive disease, n (%)	1 (5.5)	5 (6.5)	1
Dactylitis, n (%)	0 (0)	3 (4)	0.54
RF positivity, n (%)	1 (7.7)	1 (1.9)	0.36
ANA positivity, n (%)	10/19* (55.6)	12/44* (27.2)	***0.04***
Biological therapy ever, n (%)	10 (50)	63 (72.4)	0.06

SS: Sjögren’s syndrome; SD: standard deviation; RF: rheumatoid factor; ANA: anti-nuclear antibody; MRI: Magnetic resonance imaging.

* Only patients with available data were included.

## DISCUSSION

Although the development of SS is relatively common in the course of autoimmune rheumatic diseases, there is an increased awareness of the sicca symptoms and SS in association with SpA.^18,30,31^ Recent observations of Jarrot et al. are intriguing in that a substantial proportion of pSS patients have axial articular manifestations when carefully addressed. Furthermore, pSS patients in association with SpA (mostly PsA) in this study displayed HLA allele susceptibility for both pSS and SpA, suggesting that some common genetic factors could participate in the occurrence of these seemingly distinct diseases. Interestingly, this association was not relevant for PsO, and only one out of the 148 pSS patients had PsO in contrast to the 17 ASAS-classified axial SpA patients with PsO.^17^

To further investigate the lack of PsO in the prospective case series of Jarrot et al., we analysed retrospectively the frequency of sicca symptoms in both PsO and PsA. Clinical features of PsO and PsA comprise similarities. Still, their pathogenesis may incorporate deviations, and hence, arthritis.^32^ In line with the above observations in pSS, we found that the prevalence of SS in the PsA patients (PsA/SS) is substantially high, and it is significantly higher than that seen in the PsO patient group. Although we could not characterise an association with the type of articular involvement of PsA as suggested by Jarrot et al., there were two patients with isolated axial involvement and 11 others with asymmetrical articular involvement. Due to the nature of a retrospective study design, we may have missed patients with silent or atypical clinical symptoms of axial disease. Still, only one third of our PsA/SS patients had symmetrical polyarticular involvement. Disease duration, mean age, and gender distribution in both groups did not differ. Therefore, the high prevalence of SS that favors PsA in this study may indicate a shared pathogenetic background for SS and imply that these factors differ between PsO and PsA. Similarly in an earlier study, age- and sex-matched PsA patients differed significantly in reduced median maximal stimulated salivary flow rate from patients with plaque psoriasis alone, suggesting that additional pathogenetic factor operate in PsA that may be responsible for the association of PsA but not PsO with SS.^18^

Some unique pathogenetic factors that initiate arthritis in PsO might be responsible for the SS in PsA. In this regard, the unknown significance of previously reported prevalence of ANA in PsA may be of notice.^13^ We observed that PsA patients tested positive for ANA were 4.1 times more likely to have SS. Previously, a similar observation for the association of ANA with arthritis in PsO was reported by Rosenberg et al.^13^ A large study conducted by the National Institutes of Health (NIH) and a second multinational large study found the background overall ANA prevalence in healthy individuals at a serum dilution-level of 1:80 as 13.8% and 13.3%, respectively.^33,34^ More than half of the PsA/SS patients in our study were ANA positive with the majority (6/10, 60%) reaching a dilution level of 1:320 and beyond (**Table 1**). Compared to the healthy population or the baseline figure for ANA frequency of 14% in PsA patients published previously by Johnson et al., both PsA and PsA/SS patients in this study had a two and fourfold increase in ANA positivity, respectively.^12^ Thus an autoantibody-mediated pathology may be involved in some PsO patients with arthritis and/or SS. Recently, the presence of autoantigens and related autoantibodies in PsA raised the concept of a Systemic Psoriatic Disease, emphasising the autoimmune nature of this disease characterised by multiple extra-cutaneous and -articular manifestations.^35^ T and B cell infiltration and ectopic lymphoid neogenesis in synovial tissues of PsA patients as reviewed by Chimenti et al. may imply an autoantibody driven pathogenesis in PsA/SS, as well.^35^

PsA/SS patients studied had no extra-glandular systemic involvement typical for pSS and no other clinical or laboratory findings for SLE. None of them fulfill the Systemic Lupus International Collaborating Clinics classification criteria for SLE.^36^ In line with the study of Korkus D. et al., SLE is diagnosed at a much lower rate of 0.37% in PsA, although significantly higher than the observed rate of 0.15% in a randomly determined control group that was similar in age and gender and did not have any other rheumatic disease (p=0.001).^37^ The onset of PsO was relatively early, and arthritis occurred after a median gap of 14 years. SS in PsA patients in our study seems to be a clinically mild complication that is associated with arthritis since most of the patients could not recall symptom duration for sicca symptoms, and unfortunately, we could not come to a conclusion whether SS or arthritis antedates in the course of PsO. Although clinically mild, seven patients with PsA/SS had histopathologic changes on minor salivary gland biopsies compatible with SS, and the majority of ANA-positive PsA/SS patients were SS-A positive, as well. Also, 12 patients were classified as pSS according to the 2016 ACR/EULAR criteria for pSS.^26^

In our study, SS was less frequent in PsA patients treated with TNF inhibitors or non-TNF biologic agents. Although numerically higher, the tendency towards a protective effect for SS development did not reach statistical significance. Speculative due to the retrospective nature of our study, this data may suggest that intensive therapy also decelerates inflammation in exocrine glands. It is suggested cautiously by a meta-analysis that anti-inflammatory treatment with systemic drugs such as rituximab may have a discrete effect on improving lacrimal gland function.^38^ Additionally, the possible reasons for the 53.8% rate of focal lymphocytic sialadenitis in MSG histopathology in our study may be that the diagnosis was made as early as possible because the patients were already followed up in the rheumatology clinic and that the inflammatory processes were delayed due to the effect of the immunosuppressive treatments received for other rheumatic diseases. In addition, this result emphasises the need for more comprehensive histopathological studies in PsA/SS overlap patients. However, in randomised controlled trials of immunosuppressive treatments in pSS, positive effects on sicca symptoms of these therapies have not been fully shown yet.^39^

Twenty of our patients are diagnosed with SS according to the 2002 AECG criteria, 12 patients are diagnosed with SS according to the 2016 ACR/EULAR criteria. The reason for this difference is that salivary gland scintigraphy findings was a criterion in the 2002 AECG criteria, but not included as a criterion in 2016. In addition, according to the 2002 AECG criteria, positivity of any two of the items “ocular signs”, “salivary gland involvement”, and “histopathological findings” in addition to sicca symptoms “in patients with a potentially associated disease” is sufficient for the classification of secondary SS. We applied this condition because we were investigating the frequency of SS in PsO or PsA patients at baseline. For this reason, we found the frequency of SS according to the 2002 AECG to be higher than the 2016 ACR/EULAR criteria.^24,26^

The lack of healthy control and patient control groups are among the limitations of our study. However, in the literature, the prevalence of secondary SS (sSS) varies between 4% and 31% in RA, 14% and 20.5% in SSc and 6.5% and 19% in SLE.^2^ In our study, we found the frequency of SS in PsA patients to be 17.9%, a result consistent with rheumatic diseases associated with SS in the literature. We did not find SS in PsO patients. The number of PsO patients with sicca symptoms was two. This large difference in the frequency of SS and sicca symptoms between PsO and PsA seems to have eliminated the limitation caused by the small number of patients to some extent. The results we obtained in our study also emphasise the necessity of prospective observational studies regarding the relationship between PsO/PsA and SS, in order to determine the prevalence of SS more precisely in PsO and PsA.

## CONCLUSIONS

Secondary Sjögren’s syndrome seems to be part of the clinical picture in a substantial proportion of PsA patients, and its absence in PsO implies that SS is associated with arthritis of PsA. The bidirectional rate of occurrence suggests possible shared immunopathogenic mechanisms rather than a coincidental relation. A possible association of SS with axial involvement in PsA deserves further attention. The high frequency of ANA positivity suggests that B lymphocytes may have a role in the emergence of SS as part of the recently proposed Systemic Psoriatic Disease.

## CONFLICT OF INTEREST

None declared.

## FUNDING

There is no financial institution support in the study.

## ETHICAL APPROVAL

Approval for this study was obtained from the Marmara University Faculty of Medicine local ethics committee (09.2023.416) in Istanbul, Turkey.

## DATA SHARING STATEMENT

Data are available on reasonal request.

## PATIENT AND PUBLIC INVOLVEMENT

atients and/or public were not involved in the design, or conduct, or reporting, dissemination plans of this research.
